# Eutrophic status influences the impact of pesticide mixtures and predation on *Daphnia pulex* populations

**DOI:** 10.1002/ece3.7305

**Published:** 2021-03-07

**Authors:** Talles Bruno Oliveira dos Anjos, Francesco Polazzo, Alba Arenas‐Sánchez, Laura Cherta, Roberto Ascari, Sonia Migliorati, Marco Vighi, Andreu Rico

**Affiliations:** ^1^ IMDEA Water Institute Science and Technology Campus of the University of Alcalá Alcalá de Henares Spain; ^2^ University of Koblenz‐Landau Landau in der Pfalz Germany; ^3^ Department of Economics, Management and Statistics University of Milano‐Bicocca Milan Italy; ^4^ Cavanilles Institute of Biodiversity and Evolutionary Biology University of Valencia Paterna Spain

**Keywords:** Bayesian shrinkage, eutrophication, multiple stressors, pesticides, predation

## Abstract

Pesticides, nutrients, and ecological stressors such as competition or predation co‐occur in freshwater ecosystems impacted by agriculture. The extent to which combinations of these stressors affect aquatic populations and the role of nutrients availability in modulating these responses requires further understanding. In this study, we assessed how pesticides affecting different taxonomic groups and predation influence the response of *Daphnia pulex* populations under different trophic conditions. An outdoor experiment was designed following a factorial design, with the insecticide chlorpyrifos, the herbicide diuron, and the predation by *Notonecta* sp. individuals as key stressors. The single impact of each of these stressors, and their binary and tertiary combinations, was evaluated on *D. pulex* abundance and population structure under mesotrophic and eutrophic conditions for 21 days. Data were analyzed using generalized linear mixed models estimated by means of a novel Bayesian shrinkage technique. Our study shows a significant influence of each of the evaluated stressors on *D. pulex* abundance; however, the impacts of the herbicide and predation were lower under eutrophic conditions as compared to the mesotrophic ones. We found that binary stressor interactions were generally additive in the mesotrophic scenario, except for the herbicide–predation combination, which resulted in synergistic effects. The impacts of the binary stressor combinations in the eutrophic scenario were classified as antagonistic, except for the insecticide–herbicide combination, which was additive. The tertiary interaction resulted in significant effects on some sampling dates; however, these were rather antagonistic and resembled the most important binary stressor combination in each trophic scenario. Our study shows that the impact of pesticides on freshwater populations depends on the predation pressure, and demonstrates that the combined effect of pesticides and ecological stressors is influenced by the food availability and organism fitness related to the trophic status of freshwater ecosystems.

## Graphical abstract

Our study shows that the impact of pesticides on *Daphnia pulex* populations is influenced by predation. Moreover, it demonstrates that the combined effect of pesticides and ecological stressors is influenced by food availability and organism fitness related to the trophic status of freshwater ecosystems. Therefore, this study supports the incorporation of ecological interactions and trophic status as key parameters for the risk assessment of pesticides.

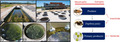

## 1 INTRODUCTION

Agriculture production is one of the most critical pathways for nutrients into aquatic ecosystems worldwide (Woodward et al., 2012). Nutrient contamination usually leads to eutrophication (Cooper, 1993), which is characterized by an increase in algae biomass and primary productivity which, in turn, modifies the structure of primary and secondary consumer communities (Bray et al., 2019; Declerck et al., 2011). Besides nutrient loads, surface waters surrounding agricultural fields also receive pesticide inputs, which can produce toxic effects on aquatic organisms and contribute to a biodiversity decline (Beketov et al., 2013; Chiu et al., 2016). Since pesticides are applied for targeting various pests in different crops simultaneously, mixtures of pesticides with different mode of action co‐occur in freshwater ecosystems (Schreiner et al., 2016). Therefore, studying the impacts of pesticide mixtures on ecosystems with different levels of eutrophication is a more realistic approach than focusing solely on one compound under pristine conditions.

Under natural conditions, indirect pesticide effects may occur as a result of biotic interactions such as competition or predation. These biotic interactions, often called natural or ecological stressors, can magnify the toxic impact of pesticides on aquatic populations and communities (Janssens & Stoks, 2013; Trekels et al., 2013). Effects of biotic interactions following pesticide exposure are not considered in the low or intermediate tiers of pesticide risk assessment (Relyea & Hoverman, 2006). However, it is expected that species interactions such as predation enhance the risks of pesticides for sensitive prey populations, resulting in additive or even synergistic effects. To date, most studies evaluating the interaction between pesticides and predation have been performed using one single chemical stressor (mainly insecticides) and under unlimited food conditions (Viaene et al., 2015; Del Arco et al., 2015; van den Brink et al., 2017), while the interaction between pesticides affecting different taxonomic groups and predation may vary according to the trophic status of ecosystems and the consequent food quantity and quality (Fleeger et al., 2003).

This study aimed to assess how pesticides affecting aquatic organisms of different trophic levels and predation influence the response of a zooplankton population under different trophic conditions. An outdoor experiment was designed with *Daphnia pulex* as focal test species and *Notonecta* sp. (Hemiptera) as predator. *D. pulex* is usually found in high abundances in a wide range of mesotrophic and eutrophic ponds and lakes (Crease et al., 2012), where they cohabit with *Notonecta* sp. and other predators (Hanazato & Dodson, 1995). The studied compounds were the organophosphorus insecticide chlorpyrifos and the phenylurea herbicide diuron. Both pesticides are included in the list of priority substances of the Water Framework Directive (WFD) (EC, 2011) and are highly relevant for European surface waters as well as globally due to their widespread use and ecological risks (Arenas‐Sánchez et al., 2019; Huang et al., 2020; Rico et al., 2021). As an acetylcholinesterase inhibitor, chlorpyrifos disturbs the signal transmission in the nervous system, causing impairment in mobility (Sharma et al., 2012) and eventually mortality, particularly on aquatic arthropods such as *D. pulex* (Cuppen et al., 2002; Daam et al., 2008). Diuron is a nonselective herbicide which acts as a competitor for electrons at the acceptor plastoquinone QB and inhibits the electron transport chain in the photosystem II, thus affecting the growth of algae and macrophytes (Jansen et al., 1993).

By testing these species and stressor combinations, we aimed to gain mechanistic understanding on the effects caused by multiple stressors on freshwater populations. We hypothesized that direct toxic effects of chlorpyrifos on *D. pulex* and the indirect effects of diuron caused by a decrease in food availability will decrease *D. pulex* fitness, increasing its population susceptibility to the predator. Moreover, we hypothesized that the susceptibility of *D. pulex* will be larger when chlorpyrifos and diuron co‐occur, and we expected to see an influence of the trophic status on the response to the single and combined effects of the evaluated pesticides.

## 2 MATERIAL AND METHODS

### 2.1 Experimental design

**FIGURE 1:**
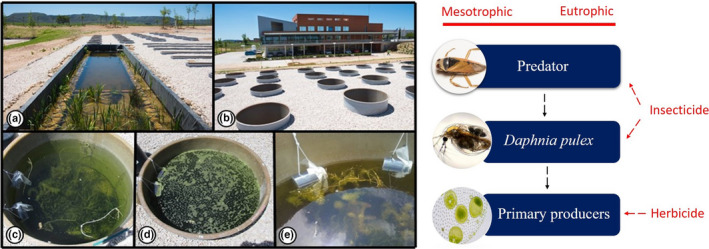
Mesocosm facilities (left) and experimental setup (right). Biodiversity lagoon used to fill in the mesocosms (a), outdoor mesocosms (b), mesotrophic mesocosms (c), eutrophic mesocosms (d), detail of experimental cages containing *Daphnia* *pulex* alone and *Daphnia* *pulex* with one individual of *Notonectidae* sp (e).

An experiment was performed by introducing two cages containing *D. pulex* individuals into each of the 24 mesocosms (850 L) of the outdoor experimental facilities of the IMDEA Water Institute (Alcalá de Henares, Spain), see Figure 1. Each cage contained 50 adults and 50 subadults/juveniles (referred as juveniles from now onwards) of *D. pulex*. The cages were built using an acrylic cylinder (length: 17 cm, diameter: 10.5 cm; volume: 1.5 L) covered on both sides by a 200 µm filter membrane (Figure 1). A piece of string was inserted on each cage, and they were fixed close to the wall of the mesocosms. The depth of the cages was continuously regulated to keep three‐quarters of the cages immersed in the mesocosm water. All cages were introduced in the mesocosms three days prior to the start of the experiment and were checked and shake regularly to prevent excessive biofilm formation in the filter membrane and to homogenize the cage exposure medium with that of the mesocosm.

The experiment was performed according to a crossed factorial design (*n* = 3), with the following treatments: chlorpyrifos (1 µg/L, two levels: presence/absence), diuron (18 µg/L, two levels: presence/absence), and predation (two levels: presence/absence), and was run in parallel under two different trophic conditions: mesotrophic and eutrophic. The selected concentration of chlorpyrifos was assumed to be highly toxic to *D. pulex* (EC50‐48h: 0.25 µg/L, van der Hoeven & Gerritsen, 1997), while *Notonecta* was expected to be more resistant to this compound based on available toxicity data (LC50‐96h, *N. maculata:* 7.97 µg/L; Giddings et al., 2014). One liter stock solution of chlorpyrifos and/or diuron (prepared with Milli‐Q water and 2 ml of methanol) was poured over the respective mesocosms on 27 May 2019 and stirred with a wooden stick. The mesocosms that did not receive chlorpyrifos and diuron also received a blank solution containing the same amount of milli‐Q water and methanol. The concentration level of methanol used in this study is usually allowed in toxicity experiments as it is expected to dissipate fast from the water column and does not cause toxic effects to invertebrates (OECD, 2000).

In each mesocosm, there were two cages, one exposed to predation and the other not. In the cages exposed to predation, a *Notonecta* sp. individual (approximately 130 mg wet weight) was introduced on the same day the pesticides were applied. *Notonecta sp*. individuals were collected from the mesocosm facilities in which the experiment was performed prior to the pesticide application. One predator was placed together with 100 *D*. *pulex* individuals inside the cages. Such prey–predator ratio was based on the numbers monitored in the mesocosms, which roughly ranged between 40 and 130. However, the experimental units used here offer a smaller volume than the actual volume in which these two species interact, which should be considered for the interpretation of the results and for their extrapolation to natural conditions.

The mesotrophic mesocosms did not receive nutrients, while the eutrophic ones were amended twice a week with a solution containing 1.820 g of NH_4_NO_3_ and 0.208 g of KH_2_PO_4_, which resulted in a concentration of 750 µg/L of N and 75 µg/L of P. The concentrations of N and P added to the eutrophic mesocosms are in accordance with the values for eutrophic aquatic ecosystems discussed by Baban (1996). Nutrients’ addition started three weeks before the application of the pesticides.

### 2.2 Pesticide sampling and analysis

Two hours after the application of the pesticides, 0.5 L of water was sampled and stored in amber glass bottles to verify initial pesticide concentrations. In addition, samples were taken on days 1, 3, 7, and 10 in the mesocosms that received the chlorpyrifos application to assess its dissipation, and on days 7 and 21 in the mesocosms that received diuron. The sampling times were decided based on the theoretical half‐lives (or dissipation time for the 50% of the compound: DT50) of these substances in water systems, which were 5 days for chlorpyrifos and 43 days for diuron (PPDB, 2020). All samples were stored at −20°C until further analyses.

The concentration of chlorpyrifos in the water samples was analyzed using a gas chromatograph (GC) system (Agilent 7890A) coupled to a mass spectrometer (MS) with a triple quadrupole analyzer (Agilent 7000 GC/MS Triple Quad). The limit of quantification (LOQ) and the limit of detection (LOD) for chlorpyrifos were 30 ng/L and 3 ng/L, respectively (Supporting Information—Annex I). Diuron was analyzed using a high‐performance liquid chromatography system (Agilent Technologies 1200) coupled to a time‐of‐flight mass spectrometer (TOF‐MS) (Agilent Technologies 6230). The LOQ and the LOD for diuron were 2000 ng/L and 600 ng/L, respectively (Supporting Information—Annex II). The DT50 for chlorpyrifos and diuron in the mesocosm water of each treatment was calculated by dividing Ln(2) by the mean dissipation coefficient. The dissipation coefficients were calculated by linear regression of the ln‐transformed measured concentrations with the software Microsoft Excel version 2010 assuming first‐order kinetics.

### 2.3 Water quality parameters

Water samples (0.5 L) were collected on days −5, 7, and 14 relative to the application of the pesticides in order to analyze the concentrations of ammonia, nitrate, ortho‐phosphate, and total P (APHA, 2005). The total inorganic N was calculated as the sum of the concentrations of nitrogen as ammonia and nitrate. Temperature, dissolved oxygen, pH, electric conductivity, and total dissolved solids (TDS) were measured using a multimeter (HANNA HI0194) in the morning (8 a.m.) and evening (7 p.m.) on days −5, 7, and 15 relative to the application of the pesticides.

Additional water samples (0.5 L) were taken on days −5, 7, and 15 to assess the concentration of chlorophyll a. Chlorophyll *a* concentrations were measured according to the method described in APHA (2005) and were used as a proxy for suspended microalgae density.

### 2.4 *Daphnia pulex* monitoring

The experimental cages were removed from the mesocosms on days 2, 7, 14, and 21 relative to the pesticides’ application, and the abundance of *D. pulex* was assessed. The number of sampling events and time interval between samplings were pragmatically decided to observe potential short‐ and long‐term effects without overstressing the test individuals. After sampling, the population was divided into age categories based on the individual's size. In this way, adults and juveniles were separated by carefully filtering the sample over a mesh size of 1,000 µm. After counting, the animals were placed back into the cages and returned to their original mesocosms. The health status and survival of the predator was monitored daily.

### 2.5 Data analyses

A three‐way analysis of variance (ANOVA) was used to test the influence of chlorpyrifos, diuron, nutrients, and their interactions on water quality parameters. Prior to that, normality and homogeneity of variances of the data were tested using the Shapiro–Wilk and Levene's tests, respectively. These analyses were performed using the R language‐based software Jamovi (The Jamovi Project, 2019). Graphs were constructed using the program SigmaPlot Version 12 (Systat Software Inc., 1735 Technology Drive, Suite 430, San Jose, CA 95110).

Generalized linear mixed models (GLMMs) with Poisson distribution and log‐link function were used to estimate how chlorpyrifos, diuron, and predation affect the *D. pulex* population under different trophic conditions (i.e. mesotrophic and eutrophic). For each day of observation (i.e., 2, 7, 14, and 21), models were built for each group of *D. pulex* individuals (i.e., adults, juveniles, and the total population). In these models, the abundance of *D. pulex* was used as dependent variable, and the stressors (i.e., chlorpyrifos, diuron, and predation) and their interactions were included as fixed effects. The models included mesocosms nested within a block as a random effect within the model attributed to the dependence of the two cages with and without the predator *Notonecta* sp. placed at the same mesocosm.

The *D. pulex* adult dataset showed a high number of zero counts on almost every combination of sampling days and trophic conditions. This led to a separation issue, which arises when a linear combination of the predictors perfectly predicts the dependent variable. A common approach to deal with the separation problem is to drop some predictors, but this can result in ignoring very important variables and in specification bias as well (Zorn, 2005). A more suitable solution is to shrink the estimates of fixed effects coefficients toward zero to stabilize them. This can be implemented taking advantage of Bayesian inference (see Jackman, 2009) which arises quite naturally in the GLMM framework, since the classical estimates based on (restricted) maximum likelihood can be expressed as Bayesian estimates with a constant prior on the fixed effects (Laird & Ware, 1982). Here, we used a weak prior, namely multivariate normal with null mean vector and diagonal covariance matrix with variances equal to 10, which is capable of producing stable, regularized estimates while still being weakly informative (Gelman et al., 2008). Moreover, we imposed a flat prior to the random‐effects covariance matrix. In this setting, it was easy to include prior information, and therefore to fulfill the estimation issue, by modifying the usual iteratively reweighted least squares (IRLS) algorithm via augmentation of the dataset (for a technical description of the method see the Supporting Information, Annex III). Once the posterior distribution had been computed on the basis of the likelihood function and of the specified prior distributions, the final estimates $\hat{\beta }$ could be obtained, as well as reliable estimates of the covariance matrix of the fixed effects, and thus of the standard errors. This matrix was computed evaluating the inverse of the second derivative matrix of the log‐posterior distribution at $\hat{\beta }$ (Gelman et al., 2013). All GLMM analyses were performed in R 4.0.1 through the “blme” package (Chung et al., 2013; R Core Team, 2020). Model terms with a P‐value lower than 0.05 were considered significant.

Binary stressor interactions were evaluated according to the classification system described by Piggott et al. (2015). Briefly, when two single stressors were statistically significant, and the interaction among them was not, the interaction was classified as additive (AD); therefore, the result of the two stressors was the sum of the individual stressor effects. Interactions that deviate from additivity, that is, showing a significant interaction term, were either classified as antagonistic (A) or synergistic (S). Positive antagonistic (+A) effects were assumed when the interaction produces an abundance increase that is lower than the effect created by the sum of both stressors, while negative antagonistic (−A) effects were assumed when the abundance decrease was lower than the addition of both stressors. Synergistic interactions were defined when the effect caused by both stressors was greater than the sum of the individual effects. Effects were classified as positive synergistic (+S) when the abundance increase was higher than that predicted by the sum of both stressors, while negative synergistic (−S) effects were classified as those for which the abundance decrease was larger than that predicted by the addition of both stressors. It is noteworthy to mention that the approach described by Piggott et al. (2015) does not apply to triple interactions, nor they are usually considered in multiple stressors research. In this study, we categorized them by inspecting the results of the statistical test and by qualitatively comparing the results with the impacts of the corresponding single and binary stressor combinations.

## 3 RESULTS

### 3.1 Pesticide concentrations

Measured concentrations of chlorpyrifos 2 hr after the pesticide application ranged between 89% and 93% of the intended dose, while the measured concentrations of diuron ranged between 106% and 121% (Table S1). Neither diuron nor chlorpyrifos residues were found in the controls at any time during the experiment, nor were traces of diuron found in mesocosms treated with chlorpyrifos only, and vice versa. The DT50 calculated for chlorpyrifos ranged between 3.4 and 1.6 days, and concentrations on day 7 after the application were below 25% of the initial concentrations, and relatively low thereafter (Table S1). The DT50 for diuron ranged between 45 and 34 days within the different treatments, so at the end of the experimental period, concentrations were above 70% of the initial concentration. For both pesticides, a slightly faster dissipation was found in eutrophic mesocosms as compared to the mesotrophic ones (Table S1), particularly after day 7.

### 3.2 Water quality parameters

**FIGURE 2:**
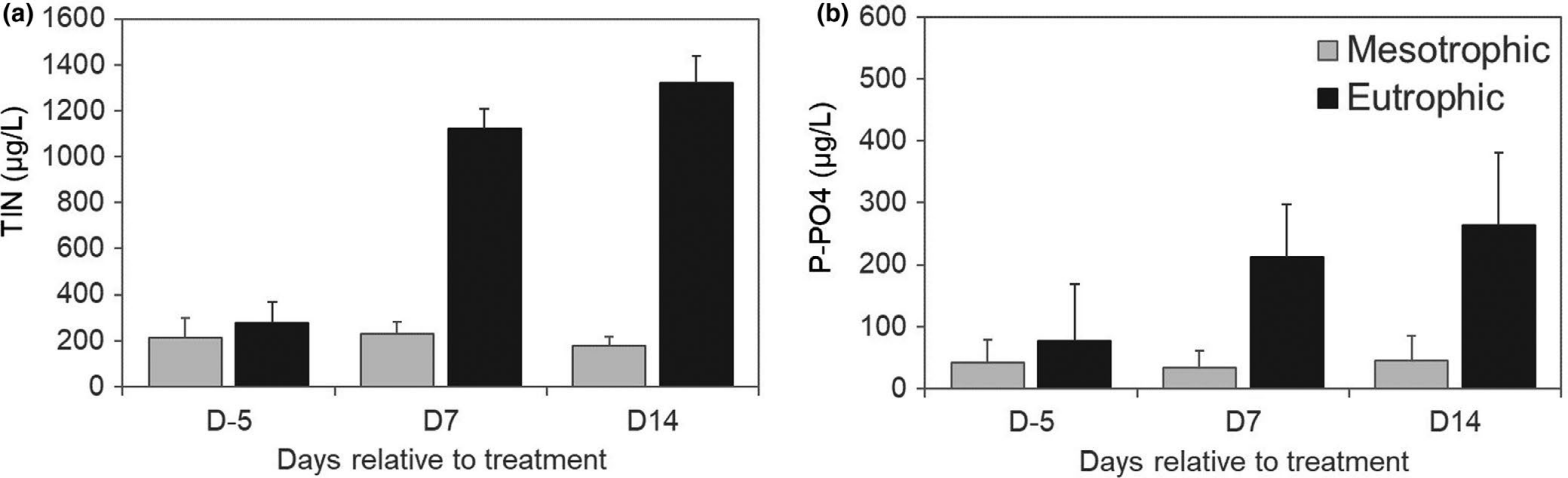
Mean concentrations of total inorganic nitrogen (a) and phophorous as ortho‐phosphate (b) measured in the mesotrophic and eutrophic mesocosms during the experiment. Error bars indicate one standard deviation

Average dissolved total inorganic nitrogen and phosphorous (as ortho‐phosphate) concentrations were 206 µg/L and 40 µg/L in the mesotrophic mesocosms, and 907 µg/L and 184 µg/L in the eutrophic ones (Figure 2). These nutrient concentrations are in the range of the classification limits for mesotrophic and eutrophic surface water ecosystems (Wetzel, 2001). The mean values for ammonia, nitrate, and ortho‐phosphate, together with the total nitrogen and total phosphorous concentrations in the different sampling days are provided in Table S2.

The water pH remained between 8.3 and 10.6 during the experiment. The exposure to the herbicide diuron resulted in a significant decrease in pH in all sampling days (Table S3). On the other hand, the addition of nutrients and the insecticide chlorpyrifos resulted in a significant increase in the water pH on days 7 and 15, respectively (Table S3). In the eutrophic mesocosms, the dissolved oxygen concentrations were significantly higher as compared to the mesotrophic ones on days −5 and 7 relative to the pesticide application. Reduction in the dissolved oxygen concentration was significant in the mesocosms treated with the herbicides on days 7 and 15 (Table S3). These responses of pH and dissolved oxygen are in accordance with the changes in primary production that may be induced by the different treatments. Electric conductivity was significantly higher in the mesocosms treated with nutrients as compared to the nontreated ones (Table S3), potentially due to the K supply from KH_2_PO_4_.

The concentration of chlorophyll a significantly increased in the mesocosms that received nutrients, becoming between 3 and 6 times higher than the concentrations measured in the mesotrophic mesocosms (Table 1). This supports the increase in primary productivity and algae biomass in the eutrophic mesocosms. The herbicide diuron did not clearly reduce or increase chlorophyll a concentrations if the temporal trend is inspected; however, the interaction between diuron and nutrients resulted in a lower chlorophyll a concentration on day 7 when compared to the mesocosms treated with nutrients only. The application of the insecticide and the rest of nutrient and pesticide interactions were not statistically significant (Table 1).

**TABLE 1:** Mean and standard deviation (*SD*) of chlorophyll a concentrations (µg/L) in the different treatments

Treatment	Days relative to the pesticides application					
	D5		D7		D15	
	Mean	*SD*	Mean	*SD*	Mean	*SD*
C	4.63	1.34	1.96	0.62	4.27	1.93
N	**28.6**	28.2	**11.2**	2.14	**12.8**	6.67
H	3.56	0.62	**3.56**	1.11	6.23	3.53
I	5.16	0.31	4.27	2.45	4.81	2.14
H‐N	16.4	1.72	**2.31**	1.11	8.19	1.63
I‐N	18.7	23.4	10.5	4.45	24.2	21.5
I‐H	3.20	0.10	4.09	3.39	19.7	27.4
I‐H‐N	22.2	23.5	3.20	1.51	10.1	4.53

Note

Significant differences of the treatments were assessed by the three‐way ANOVA. Significant effects (*p* < 0.05) are shown in bold.

Abbreviations: C, control; H, herbicide = diuron; I, insecticide = chlorpyrifos; N, nutrients.

### 3.3 *Daphnia pulex* population responses

**FIGURE 3:**
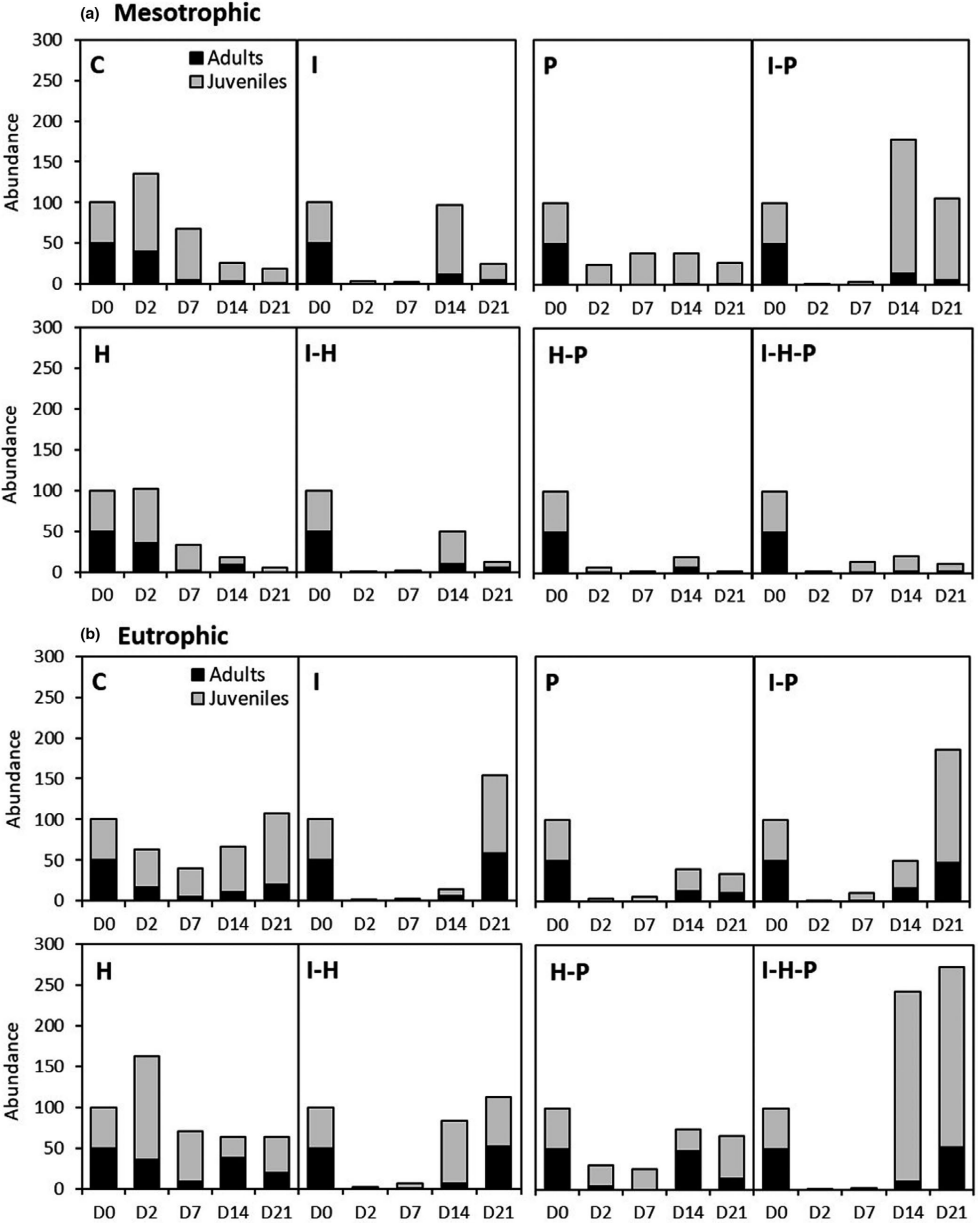
*Daphnia pulex* population abundance as response to the single and combined effects of the evaluated stressors in the mesotrophic (a) and eutrophic (b) scenario. I, insecticide = chlorpyrifos; H, herbicide = diuron; P, predation. Raw data are provided in Table S6

The meta‐population dynamics assessed in this study are intrinsically related to the life cycle of the test organisms. Under the tested conditions, *D. pulex* reproduced by parthenogenesis and usually started to produce eggs 7–10 days after they are born (Spitze et al., 1991). Based on the population structure at the start of the experiment, we expected some adults to perish some days to weeks after the start of the experiment, and an increase in juveniles between day 7 and 14 as a result of the first generations of the juveniles that were initially introduced (at least in the experimental controls). As it can be observed in Figure 3, the total population abundance declined over the course of the experiment under mesotrophic conditions, potentially due to a limitation of resources. Under eutrophic conditions, it went down initially but increased again after the second week of the experiment, reaching a higher system's carrying capacity than the mesotrophic ones (Figure 3), which is in accordance with our expectations given the larger food availability in these systems.

The application of the insecticide chlorpyrifos drastically reduced the total *D. pulex* population abundance on days 2 and 7 under the mesotrophic and the eutrophic conditions (Table 2, Figure 3). Practically, all adults were eliminated, while only very few juveniles remained (Table S4, Figure 3). On day 14, in the mesotrophic mesocosms, the total population abundance of *D. pulex* was significantly higher than in the controls, triggered by an increase in adults and juveniles, indicating population recovery (Table S4 and S5, Figure 3). However, in the eutrophic mesocosms, the negative effect on the total population abundance persisted on day 14, influenced by a relatively small number of juveniles (Table 2). On day 21, the effects of chlorpyrifos were not significant in any of the evaluated trophic scenarios (Table 2, Figure 3).

**TABLE 2:** Results of the generalized linear mixed model for *Daphnia pulex* total abundance in the mesotrophic and eutrophic scenarios

Days	Mesotrophic			Eutrophic		
	Fixed effects	$\hat{\beta }$(±*SE*)	*p*‐Value	Fixed effects	$\hat{\beta }$(±*SE*)	*p*‐Value
D2	Constant	**4.886 ± 0.119**	**<.001**	Constant	**3.952 ± 0.368**	**<.001**
	I	**−3.713 ± 0.350**	**<.001**	I	**−3.883 ± 0.704**	**<.001**
	H	−0.285 ± 0.170	.094	H	0.796 ± 0.511	.119
	P	**−1.704 ± 0.126**	**<.001**	P	**−2.708 ± 0.286**	**<.001**
	I × H	−1.088 ± 0.710	.126	I × H	−0.532 ± 0.951	.576
	I × P	−0.241 ± 0.843	.775	I × P	**1.971 ± 0.886**	**.026**
	H × P	**−1.104 ± 0.269**	**<.001**	H × P	**1.025 ± 0.307**	**<.001**
	I × H × P	**3.859 ± 1.099**	**<.001**	I × H × P	−0.768 ± 1.099	.485
D7	Constant	**3.935 ± 0.513**	**<.001**	Constant	**3.479 ± 0.459**	**<.001**
	I	**−2.825 ± 0.773**	**<.001**	I	**−2.823 ± 0.711**	**<.001**
	H	**−1.512 ± 0.726**	**.037**	H	0.694 ± 0.635	.274
	P	**−0.590 ± 0.117**	**<.001**	P	**−1.846 ± 0.245**	**<.001**
	I × H	1.068 ± 1.083	.324	I × H	0.179 ± 0.969	.854
	I × P	0.627 ± 0.468	.181	I × P	**2.999 ± 0.429**	**<.001**
	H × P	**−2.489 ± 0.476**	**<.001**	H × P	**0.818 ± 0.278**	**.003**
	I × H × P	**4.261 ± 0.765**	**<.001**	I × H × P	**−3.108 ± 0.630**	**<.001**
D14	Constant	**3.178 ± 0.381**	**<.001**	Constant	**4.110 ± 0.262**	**<.001**
	I	**1.167 ± 0.527**	**.027**	I	**−1.498 ± 0.391**	**<.001**
	H	−0.375 ± 0.540	.487	H	−0.163 ± 0.370	.661
	P	**0.405 ± 0.146**	**.006**	P	**−0.502 ± 0.115**	**<.001**
	I × H	−0.358 ± 0.746	.632	I × H	**1.953 ± 0.535**	**<.001**
	I × P	0.207 ± 0.163	.203	I × P	**1.714 ± 0.204**	**<.001**
	H × P	−0.390 ± 0.236	.099	H × P	**0.635 ± 0.151**	**<.001**
	I × H × P	**−1.110 ± 0.288**	**<.001**	I × H × P	**−0.778 ± 0.238**	**.001**
D21	Constant	**2.811 ± 0.373**	**<.001**	Constant	**4.635 ± 0.263**	**<.001**
	I	0.039 ± 0.521	.940	I	0.074 ± 0.370	.842
	H	**−1.233 ± 0.562**	**.028**	H	−0.481 ± 0.373	.197
	P	**0.361 ± 0.172**	**.036**	P	**−1.165 ± 0.114**	**<.001**
	I × H	0.888 ± 0.762	.244	I × H	0.169 ± 0.523	.747
	I × P	**1.065 ± 0.214**	**<.001**	I × P	**1.485 ± 0.131**	**<.001**
	H × P	**−1.817 ± 0.543**	**<.001**	H × P	**1.189 ± 0.152**	**<.001**
	I × H × P	0.255 ± 0.600	.671	I × H × P	**−0.457 ± 0.179**	**.011**

Note

The effect estimate ($\hat{\beta }$) indicates the magnitude and sign of the contribution of each single stressor or stressor combination to the total population abundance, while the *p*‐value indicates whether this stressor or stressor interaction is significant or not. Bold values indicate significant effects (*p*‐value < 0.05). “x” indicates stressor interactions.

Abbreviations: H, herbicide = diuron; I, insecticide = chlorpyrifos; P: predation; *SE*, standard error.

The application of the herbicide diuron resulted in a decline of the *D. pulex* population in the mesotrophic scenario, which was statistically significant on days 7 and 21 (Table 2, Figure 3). In the eutrophic scenario, however, the effect of diuron on the total population abundance was not significant. Under eutrophic conditions, diuron resulted in a significant increase in adults on day 2 and a decrease in juveniles on day 14 (Tables S4 and S5, Figure 3). However, the magnitude of these effects on the population structure was mild.

Predation resulted in a significant decline of the total *D. pulex* population abundance during the whole experimental period in the mesotrophic and in the eutrophic scenario (Table 2). The population decline was larger during the first week of the experiment in the eutrophic scenario. In both scenarios, there was a significant decline of adults and juveniles, although the effect on adults was larger, being eliminated in the mesotrophic scenario and significantly reduced in the eutrophic one (Tables S4 and S5, Figure 3).

The interaction between chlorpyrifos and diuron on the total *D. pulex* abundance was not significant in most of the cases. In the first week, chlorpyrifos alone practically extinguished the *D. pulex* population, so that the additional effect of the herbicide could not be properly assessed. After that, the interaction between both stressors was classified as additive. The exception was the significant interaction on day 14 in the eutrophic scenario, triggered by an increase in juveniles (Table S4), which was classified as negative antagonistic (‐A). However, on day 21 such interaction was not significant.

The combined effect of chlorpyrifos and predation on the total *D. pulex* population in the mesotrophic scenario was mainly driven by the effects of chlorpyrifos and resulted in additive effects on days 2, 7, and 14, while on day 21 there was a significant population increase (mainly due to the increase in juveniles; Figure 3, Table S4), which was classified as positive synergistic (+S). In the eutrophic scenario, it followed a very similar trend. However, the interaction between chlorpyrifos and predation was found to be statistically significant in all sampling days (Table 2). Such interaction resulted in an increase in the number of individuals as compared to the predation treatment alone and was classified as negative antagonistic (−A).

The combined effects of diuron and predation on *D. pulex* abundance resulted in significant effects in most sampling days in both trophic scenarios (Table 2). In the mesotrophic scenario, the interaction between both stress factors resulted in a population decline, which was larger than the sum of both single stressors and was classified as negative synergistic (−S). However, in the eutrophic mesocosms the *D. pulex* abundance decrease was lower than in the predation treatment and the interaction was classified as negative antagonistic (−A).

The interaction between the three stressors was significant in several sampling days in both trophic scenarios (Table 2). In the mesotrophic scenario, the effect estimate ($\hat{\beta }$) for total abundance was positive on days 2 and 7, and negative on day 14 (Table 2). The population response, however, was very similar to that in the herbicide–predation cages. In the eutrophic scenario, the triple interaction was significant on days 7, 14, and 21, showing a negative beta estimated effect triggered by the reduction of juveniles (Table S4, Figure 3). In this case, the decline in total abundance related to the interaction among these three stressors resembled that observed in the insecticide–predation treatment (Figure 3).

## 4 DISCUSSION

Our study shows a significant *D. pulex* population decline related to the individual effect of each of the evaluated stressors. Chlorpyrifos resulted in a fast population decline, which was expected based on the toxicity of this compound (van der Hoeven & Gerritsen, 1997). The magnitude of the population decline was similar between both trophic scenarios, indicating a similar population sensitivity (Table 2). After the compound had dissipated from the water column, there were opportunities for recovery based on the reproduction capacity of the few surviving individuals. The population recovery patterns were slightly different between the evaluated scenarios, being faster under mesotrophic conditions but declining again on day 21. On the other hand, under eutrophic conditions, the maximum recovery potential was observed on day 21. In principle, a faster recovery would have been expected in the eutrophic scenario, since we recorded a slightly faster dissipation of chlorpyrifos (Table S1), which can be attributed to differences higher microbial degradation and phytoplankton uptake (Racke, 1993). Moreover, the eutrophic scenarios held a higher primary productivity, which was expected to enhance growth of the surviving organisms and contribute to a larger reproductive success (Wuerthner et al., 2019). A possible explanation for the observed delayed recovery in this scenario is the accumulation of chlorpyrifos in algae (Lal et al., 1987), and the continued uptake by *D. pulex* after dissipation of the dissolved fraction from the water column. Our study also shows that the *D. pulex* population previously exposed to the insecticide had a significant increase in abundance toward the end of experiment, after the compound had dissipated from the water column. This may be partly related to an increase in the brood size due to a reduction in intraspecific competition inside the cage (Liess & Foit, 2010), but also to an evolutionary response to improve recovery capability when a stress factor disappears, as discussed by Kimberly and Salice (2015).

The application of diuron resulted in a moderate, but statistically significant, long‐term decrease in the *D. pulex* population in the mesotrophic scenario. This could be related to the decrease in the amount and quality of phytoplankton, as chronic toxicity of diuron to *D. pulex* has been reported to be orders of magnitude higher than the tested concentration (LOEC 7.7 mg/L, Nebeker & Schuytema, 1998). In a previous mesocosm experiment performed with diuron, Hasenbein et al. (2017) observed a decrease in phytoplankton richness and an increase in grazing‐resistant algal species, which support our explanation. Moreover, the mesocosms treated with diuron, although did not show a statistically significant decrease in chlorophyll a concentration (Table 1), showed a significant reduction in the pH, conductivity, and dissolved oxygen concentration (Table S3), which indicate a reduction in the primary productivity and biomass. In our study, the indirect effects caused by diuron on *D. pulex* were not observed in the eutrophic mesocosms. In this scenario, diuron effects on primary producer's biomass were probably milder than in the mesotrophic scenario, indicating that nutrients enrichment can counteract the food restrictions caused by diuron exposure and prevent indirect effects on the *D. pulex* population.

The predator *Notonecta* sp. produced a significant reduction in the *D. pulex* population abundance in both trophic scenarios at the beginning of the experimental period. *Notonecta* sp. consumed up to 10–30 individuals in one single day. In the two following weeks, the *D. pulex* population started to slightly increase again (Table 2), with a relative increase in the number of juveniles (Figure 3). As we noted in this experiment, *Notonecta* sp. had a higher feeding efficiency toward adults. This is the first time that such a feeding selective capacity is documented for *Notonecta* sp, but other experiments with freshwater predators such as *Chaoborus* sp. also describe a preference for adult and subadult organisms of *D. pulex* in similar competition experiments (Van den Brink et al., 2017). Larger organisms can be more easily hunted as their swimming velocity is lower. It should be noted, however, that the small size of the systems and the unavailability of refugees could have enhanced the encounter rates between the predator and its prey. In this way, the feeding rates shown here may be considered conservative estimates of actual feeding rates observed in nature, the more since *Notonecta* sp. is expected to have a more varied diet than feeding solely on *D. pulex*.

Overall, most studies assessing the combined effects of insecticides and herbicides on freshwater populations and communities describe additive effects (Rodney et al., 2013). A mesocosm study performed by Relyea (2009) with a single application of five herbicides and five insecticides (including chlorpyrifos) demonstrated that in many cases the effects of the mixtures on zooplankton communities were dominated by those imposed by the insecticide. Choung et al. (2013) found that the presence of the herbicide atrazine did not influence the impact of the insecticide terbufos on cladocerans under microcosm conditions. In line with this, our study shows that the combined impacts of chlorpyrifos and diuron were additive, and were triggered by the toxic effect exerted by the insecticide. Such additive effects were recorded during the time in which both stressors co‐occurred, but also persisted after chlorpyrifos had dissipated from the water medium. This indicates that at the tested exposure levels of chlorpyrifos, the lethal and long‐term reproductive effects on *D. pulex* play a much more important role than the indirect effects caused by diuron. This was expected for the eutrophic scenario, as we found insignificant indirect effects of diuron in the *D. pulex* population, but we have demonstrated that this also holds true for mesotrophic systems, and potentially also for most oligotrophic systems based on the literature review performed by Rodney et al. (2013).

The effects of chlorpyrifos and predation on *D. pulex* were primarily additive in the mesotrophic scenario and antagonistic in the eutrophic one. According to Fleeger et al. (2003), non‐additive responses between these stressors can occur when the insecticide increases the susceptibility of the prey by affecting predator's avoidance, and/or when the insecticide affects the feeding efficiency and the hunting capacity of the predator. In our study, antagonism was more clearly observed toward the end of the experimental period, when the insecticide had dissipated from the water column. It was potentially related to partial effects on the mobility and the hunting capacity of the predator due to toxicant accumulation. Similarly, the laboratory studies performed by Viaene et al. (2015) and Van den Brink et al. (2017) describe effects on the hunting efficiency of *Chaoborus* sp. on *D. magna* after exposure to pyrene and chlorpyrifos, respectively, which resulted in larger population abundances as compared to the treatments that were only affected by predation. These studies also discuss that population structure may be significantly affected by the combination of both co‐occurring stressors, and identified a reduction in the number of adults due to slower mobility and predator avoidance during the chemical exposure period. Changes in population structure are important as might reduce the population's resilience against other toxicants and natural stressors (e.g., by affecting intraspecific competition, Gergs et al., 2013), and can induce trophic chain effects by affecting algae grazing efficiencies and food provision for predators (Englert et al., 2012; Rodrigues et al., 2018). In our study, effects on the population structure caused by the predator were observed and were similar to those described elsewhere (Coors & de Meester, 2008; Swift, 1992). However, these did not clearly differ in the treatments that were also affected by chlorpyrifos, at least during the first period of the experiment, due to the high impact of the insecticide on *D. pulex* abundance. After the insecticide had dissipated, the reduced hunting capacity of the predator allowed the growth and reproduction of few adults, thus allowing an increase in the *D. pulex* abundance as compared to the predator treatment. Therefore, the findings of our study, together with the available literature, confirm that the effects of chemicals and predators on population abundance and structure are a result of the magnitude of the pesticide toxic pressure, the relative sensitivity of the prey and the predator, and their individual and population postexposure recovery capacity.

This is one of the first studies investigating the combined effects of an herbicide and predation on aquatic populations. The interaction between these two stressors resulted in negative synergistic effects on the *D. pulex* abundance in the mesotrophic scenario, while in the eutrophic one the effects were antagonistic. The reduction of food availability caused by diuron in the mesotrophic scenario could have contributed to the exhaustion of energy reserves, reducing *D. pulex* mobility and their predator scaping capacity (despite the small size of the cages), explaining the differences between the population decline in the predator and the herbicide‐only treatments. The mechanisms behind the antagonistic response observed in the eutrophic systems are, however, less clear. One possible explanation for such antagonistic interaction is the large asymmetry between the single effect sizes of the two evaluated stressors, being the impact of the herbicide in this case too weak. As discussed by Jackson et al. (2016), this is the main cause for most of the antagonistic responses observed in nature and occurs when the magnitude of the worst stressor completely overrides the effect of the weaker one, thereby negating its contribution to the net impact. However, in order to elucidate the mechanisms explaining the interactions between indirect herbicide effects and predators on freshwater herbivorous, further experiments should focus on quantifying the influence of food resource availability and predator avoidance on the energy budgets of these organisms.

As discussed by several authors, the combined effects of pesticides and predation depend on the sensitivity of the species to each chemical, and their fitness and reproductive capacity (Hanazato & Dodson, 1995; Vighi & Rico, 2018). In our study, we found significant *D. pulex* population effects caused by the interaction between the three evaluated stressors. However, the population abundance declines were not very different or even lower than those observed in the herbicide–predation treatment in the mesotrophic mesocosms, or the insecticide–predation treatment in the eutrophic ones. Therefore, it seems that the triple interaction effectively resembled the impact of the dominating binary stressor combination under each trophic condition, demonstrating that trophic conditions and single stressor intensity influences the sign and the magnitude of complex stressor mixtures.

## 5 CONCLUSIONS

This study shows that predation influences the response *D. pulex* populations to insecticide and herbicide contamination. Furthermore, it shows that the combined effects of the insecticide and the herbicide, or the combined effects of the insecticide and predation, are generally additive under mesotrophic conditions, while the interaction between the herbicide and predation results in synergistic effects, potentially due to lower food availability and higher susceptibility of the prey. Different results were observed under eutrophic conditions, where antagonistic responses tend to dominate because of larger food availability and enhanced population fitness (limiting the effects of one single stressor). The interaction between the three stressors was significant in both trophic scenarios; however, their impact was found to resemble that of the most dominant binary stressor combination in each case. This study demonstrates that the risk assessment of pesticides on aquatic populations should consider the trophic status of aquatic ecosystems and prey–predator relationships, and provides mechanistic understanding for predicting multiple stressor effects in aquatic ecosystems.

## CONFLICT OF INTEREST

The authors of this study declare no conflicts of interest.

## AUTHOR CONTRIBUTION

**Talles Bruno Oliveira dos Anjos:** Conceptualization (equal); Formal analysis (equal); Investigation (equal); Writing‐original draft (equal). **Francesco Polazzo:** Conceptualization (equal); Formal analysis (equal); Investigation (equal); Writing‐review & editing (equal). **Alba Arenas‐Sánchez:** Investigation (equal); Writing‐review & editing (equal). **Laura Cherta:** Formal analysis (equal); Investigation (equal); Writing‐review & editing (equal). **Roberto Ascari:** Formal analysis (equal); Methodology (equal); Writing‐review & editing (equal). **Sonia Migliorati:** Formal analysis (equal); Methodology (equal); Writing‐review & editing (equal). **Marco Vighi:** Conceptualization (equal); Investigation (equal); Supervision (equal); Writing‐review & editing (equal). **Andreu Rico:** Conceptualization (equal); Investigation (equal); Project administration (equal); Supervision (equal); Writing‐review & editing (equal).

## ETHICS STATEMENT

No ethical issues have been aroused by this study.

## Supporting information

Supplementary Material

## Data Availability

The Supporting Information file contains the raw data generated by this study and can be found at the Dryad Digital Repository https://doi.org/10.5061/dryad.qv9s4mwdb.
