# Supplementary material for: Eutrophic status influences the impact of pesticide mixtures and predation on Daphnia pulex populations
Source: Ecol Evol. 2021 Mar 7;11(9):4046–57. doi: 10.1002/ece3.7305 (PMC8093730; doi:10.1002/ece3.7305)
Supplement: Supplementary file 1 — Supplementary Material [file ECE3-11-4046-s001.docx]

**Supporting Information**

**Eutrophic status influences the impact of pesticides mixture and predation on *Daphnia pulex* populations**

Talles Bruno Oliveira dos Anjos^1,2^, Francesco Polazzo^1^, Alba Arenas-Sánchez^1^, Laura Cherta^1^, Roberto Ascari^3^, Sonia Migliorati^3^, Marco Vighi^1^, Andreu Rico^1^

^1^ IMDEA Water Institute, Science and Technology Campus of the University of Alcalá, Avenida Punto Com 2, 28805, Alcalá de Henares, Madrid, Spain.

^2^ University of Koblenz-Landau, Fortstraße 7, 76829 Landau in der Pfalz, Germany.

^3^ Department of Economics, Management and Statistics, University of Milano-Bicocca, via Bicocca degli Arcimboldi 8, 20126 Milan, Italy.

**Annex I:** Analytical method for the determination of the insecticide chlorpyrifos

**Instrumentation**

A gas chromatograph (GC) system (Agilent 7890A) coupled to a mass spectrometer (MS) with a triple quadrupole analyzer (Agilent 7000 GC/MS Triple Quad) was used for chlorpyrifos analysis. The system was equipped with an automatic multipurpose autosampler (Gerstel), a thermal desorption unit (TDU) and a cooled injection system (CIS).

The GC column used was a HP-5ms capillary column (30m length x 0.25 mm i.d. x 0.25 µm film thickness) (Agilent). Helium was used as carrier gas at a constant flow of 1.2 mL/min. Injection (analytes retained in Twisters) was carried out under programmable temperature vaporizing (PTV) mode by applying consecutive temperature rates in the TDU and the CIS units: 120 ºC/min from 40 ºC until 280 ºC (maintained during 8 min), and 12 ºC/s from -40 ºC until 280 ºC, respectively. The oven temperature was programmed as follows: 70 ºC (2 min); 20 ºC/min until 270 ºC; 50 ºC/min until 300 ºC (2min). Interface temperature was set to 250 ºC.

Mass spectrometer operated under electron ionization mode (EI), and the source temperature was maintained at 250 Cº. The acquisition was performed under Selected Reaction Monitoring (SRM). The following table shows the transitions selected for the determination of chlorpyrifos (a quantitative, Q, and a qualitative transition, q), as well as the corresponding chromatographic time, the collision energy (CE) for each transition and the dwell time applied for the acquisition. An internal standard (clorpirifos-d10) was used to correct possible losses along the overall procedure and/or instrumental deviations.

***GC-MS conditions of the compounds included in the method:**

| **Compound** | **Retention time (min)** | **Q (*m/z*), CE (eV)** | **q (*m/z*), CE (eV)** | **Dwell time (ms)** |
| --- | --- | --- | --- | --- |
| Chlorpyrifos | 11.309 | 197>107, 40 | 197>170, 10 | 75 |
| Chlorpyrifos-d10 | 11.276 | 198>170, 20 | 324>260, 10 | 75 |

**Sample treatment**

Sample treatment for the extraction of chlorpyrifos was based on Stir Bar Sorptive Extraction (SBSE) or *Twister* extraction. The Twister was placed in an Erlenmeyer flask with 100 mL of the water sample containing the internal standard (20 µL of a standard solution of 250 ng/mL) and stirred at 850 rpm overnight. After extraction the stir bar was introduced in a glass thermal desorption tube, placed in the thermal desorption unit of the GC-MS and thermally desorbed as indicated in the previous section.

**Accuracy and precision**

Accuracy was estimated from recovery experiments, analyzing 3 replicates of both matrices under study (i.e., mesocosm water) spiked at two levels: 30 and 100 ng/L. Precision was expressed as repeatability in terms of relative standard deviation (RSD) calculated for each fortification level.

|  | **30 ng/L** | | **100 ng/L** | |
| --- | --- | --- | --- | --- |
| Matrix | Recovery (%) | RSD (%) | Recovery (%) | RSD (%) |
| Mesotrophic water | 99 | 4 | 104 | 1 |
| Eutrophic water | 98 | 2 | 105 | 5 |

**Annex II:** Analytical method for the determination of the herbicide diuron

**Instrumentation**

Diuron analysis was performed on an HPLC system (Agilent Technologies 1200) coupled with a TOF-MS (Agilent Technologies 6230). Diuron was separated on a Kinetex 3 µm ACE C18-PFP column (50 x 2.1 mm, Symta). The specific HPLC/TOF-MS conditions are indicated in the following table:

| **TOF-MS parameters** | |
| --- | --- |
| Ionization mode | Positive |
| Sheath gas temperature | 250 °C |
| Sheath gas flow | 11 L/min |
| Drying gas temperature | 250 °C |
| Drying gas flow | 13 L/min |
| Nebulizer pressure | 25 psi |
| Capillary voltage | 4000 V |
| Nozzle voltage | 0 V |
| Skimmer | 65 V |
| Octapole | 750 V |
| Fragmentor Voltage | 75 V |
| Acquisition range | 50-1000 *m/z* |
| Rate | 1 spectrum/s |
| Time | 1000 ms/spectrum |
| **Chromatographic parameters** | |
| Mobile phases | A: 0.1% formic acid in water |
|  | B: 0.1% formic acid in acetonitrile |
| Gradient | 0 min – 5%B + 95%A |
|  | 1 min – 5%B + 95%A |
|  | 13 min – 44%B + 56%A |
|  | 14 min – 44%B + 56%A |
|  | 17 min – 100%B + 0%A |
|  | 18 min – 100%B + 0%A |
| Flow rate | 0.5 mL/min |
| Column temperature | 40 °C |
| Injection volume | 50 µL |

***HPLC-MS conditions:**

| **Compound** | **Retention time (min)** | **Formula** | **Ionization** | **Q (*m/z*)** |
| --- | --- | --- | --- | --- |
| Diuron | 11.900 | C9H10Cl2N2O | [M+H]^+^ | 233.0248 |
| Diuron-d6 | 11.850 | C9H4D6Cl2N2O | [M+H]^+^ | 239.0625 |

**Sample treatment**

Samples were centrifuged at 13000 rpm during 10 min. 20 µL of a solution of diuron-d6 of 250 ng/mL were added to 500 µL of sample and diluted with 480 µl of milli-Q water. The diluted sample was injected directly into the HPLC system.

**Accuracy and precision**

Accuracy was estimated from recovery experiments, analyzing 3 replicates of the matrix spiked at two levels, 2 and 20 ng/mL. Precision was expressed as repeatability in terms of relative standard deviation (RSD) calculated for each fortification level.

|  | **2 ng/mL** | | **20 ng/mL** | |
| --- | --- | --- | --- | --- |
| Matrix | Recovery (%) | RSD (%) | Recovery (%) | RSD (%) |
| Mesotrophic water | 100 | 4 | 101 | 1 |
| Eutrophic water | 100 | 4 | 101 | 1 |

**Annex III**: Methodological description on the estimation of GLMMs in the presence of data separation

Within a Bayesian framework, the choice of the prior distributions is a fundamental step. In the present context characterized by data separation, a well-established weak prior to $J$ fixed effects is a $J$-dimensional multivariate normal with null mean vector and diagonal covariance matrix with variances equal to 10 (Gelman et al. 2008). It is then easy to include prior information and to estimate fixed effects modifying the iteratively reweighted least squares (IRLS) algorithm by augmenting the dataset. In particular, the estimation procedure can be expressed as a weighted linear regression on observations $z_{*}= \binom{z}{\mu_{\beta}}$, predictors $X_{*}= \binom{X}{I_{J}}$ and weight vector $w_{*}=\left( \sigma_{z}^{2}, \sigma_{\beta}^{2} \right)^{-1}$, where $z$ and $\sigma_{z}^{2}$ are the pseudo-data and pseudo variances in the standard GLM formulation (McCullagh and Nelder 1989), $\mu_{\beta}$ and $\sigma_{\beta}^{2}$are vectors containing the prior means and variances (i.e., the null vector and a vector with elements equal to 10), and $X$ and $I_{J}$ are the design matrix and the $J J$ identity matrix, respectively.

This weighted regression can be handled through the usual IRLS algorithm, leading to the estimate $\hat{\beta}$. As for the covariance matrix of the fixed effects $\Sigma_{\beta},$ once the whole posterior distribution of $\beta$ has been computed on the basis of the prior distribution and of the likelihood function, a reliable approximation is given by the inverse of the second derivative matrix of its logarithm. The final estimates of the standard errors can be computed by evaluating this latter matrix at $\hat{\beta}$.

**References**

Gelman, A., Carlin, J.B., Stern, H.S., Dunson, D.B., Vehtari, A., Rubin, D.B., 2013. Bayesian Data Analysis, Third Edition. CRC Press. DOI: [10.1201/b16018](https://doi.org/10.1201/b16018)

McCullagh, P., Nelder, J.A., 1982. Generalized Linear Models. Taylor & Francis Ltd.

**Table S1.** Measured concentrations (µg/L) of chlorpyrifos and diuron in the different treatments. The values are expressed as mean concentrations calculated for the three mesocosms that received the pesticide with the calculated standard deviation between brackets. Day 0 indicates the concentrations measured 2h after the pesticides application. Intended concentrations were 1 µg/L for chlorpyrifos and 18 µg/L for diuron. I: insecticide =chlorpyrifos, H: herbicide=diuron, N: nutrients.

| **Treatment** | **Day 0** | **Day 1** | **Day 3** | **Day 7** | **Day 10** | **Day 21** |
| --- | --- | --- | --- | --- | --- | --- |
| **Chlorpyrifos** |  |  |  |  |  |  |
| I | 0.907 (±0.04) | 0.583 (±0.01) | 0.353(±0.04) | 0.122 (±0.02) | 0.045 (±0.01) | - |
| I - H | 0.923 (±0.04) | 0.560 (±0.05) | 0.416 (±0.04) | 0.158 (±0.01) | 0.098 (±0.01) | - |
| I - N | 0.892 (±0.06) | 0.519 (±0.01) | 0.318 (±0.07) | 0.051(±0.01) | 0.014 (±0.00) | - |
| I - H - N | 0.932 (±0.06) | 0.557 (±0.03) | 0.342 (±0.02) | 0.109 (0.01) | 0.053 (0.02) | - |
| **Diuron** |  |  |  |  |  |  |
| H | 21.8 (±0.83) | - | - | 16.7 (±0.59) | - | 15.2 (±0.99) |
| I - H | 20.9 (±0.35) | - | - | 17.6 (±0.62) | - | 15.8 (±0.68) |
| H - N | 20.5 (±0.31) | - | - | 15.7 (±0.15) | - | 12.1 (±0.66) |
| I - H - N | 19.1 (±0.34) | - | - | 16.1 (±0.16) | - | 14.1 (±0.40) |

**Table S2**. Mean and standard deviation (s.d.) for total N, total P, ammonia, nitrate and ortho-phosphate during the experimental period. LD: limit of detection.

|  | Day -5 | | | | |  | Day 7 | | | | |  | Day 14 | | | | |
| --- | --- | --- | --- | --- | --- | --- | --- | --- | --- | --- | --- | --- | --- | --- | --- | --- | --- |
|  | mesotrophic | |  | eutrophic | |  | mesotrophic | |  | eutrophic | |  | mesotrophic | |  | eutrophic | |
|  | mean | s.d. |  | mean | s.d. |  | mean | s.d. |  | Mean | s.d. |  | mean | s.d. |  | mean | s.d. |
| N inorg.. tot (mg/L) | 0.21 | 0.09 |  | 0.28 | 0.09 |  | 0.23 | 0.06 |  | 1.12 | 0.16 |  | 0.18 | 0.04 |  | 1.32 | 0.2 |
| P tot (µg/L) | 72.3 | 58.6 |  | 136 | 70.6 |  | 55.6 | 30.5 |  | 293 | 118 |  | 73.2 | 45.8 |  | 327 | 116 |
| N-NH_4_ (mg/L) | 0.05 | 0.06 |  | 0.04 | 0.02 |  | 0.02 | 0.05 |  | 0.28 | 0.08 |  | <LD | <LD |  | 0.5 | 0.12 |
| N-NO_3_ (mg/L) | 0.16 | 0.04 |  | 0.24 | 0.09 |  | 0.21 | 0.02 |  | 0.85 | 0.11 |  | 0.18 | 0.04 |  | 0.83 | 0.11 |
| P-PO_4_ (µg/L) | 41.2 | 38.5 |  | 77.6 | 54.8 |  | 33.8 | 28.3 |  | 212 | 88.5 |  | 44.4 | 44.6 |  | 264 | 122 |

**Table S3.** Mean and standard deviation (s.d.) for Temperature: Temp. (°C); Dissolved oxygen: DO (mg/L); Oxygen saturation: DO (%), pH (-); Conductivity: EC (µs/cm) and Total Dissolved Solids: TDS (ppm), Total Inorganic Nitrogen: TIN (mg/L), orto-phosphate: P-PO4 (µg/L) and Total Phosphorus: TP (µg/L). C: control, N: nutrients, H: herbicide, I: insecticide. Significant effects (p-value < 0.05) as result of the three-way ANOVA are indicated in bold.

| D-5 | | | | | | | | | | | | | | | | | | |
| --- | --- | --- | --- | --- | --- | --- | --- | --- | --- | --- | --- | --- | --- | --- | --- | --- | --- | --- |
|  | Temp. (°C) | | DO (mg/L) | | DO (% sat.) | | pH (-) | | EC (µs/cm) | | TDS (ppm) | | TIN (mg/L) | | P-PO_4_ (µg/L) | | TP (µg/L) | |
|  | mean | s.d. | mean | s.d. | mean | s.d. | mean | s.d. | mean | s.d. | mean | s.d. | mean | s.d. | mean | s.d. | mean | s.d. |
| C | 17.04 | 0.07 | 14.68 | 0.33 | 163.4 | 5.50 | 9.69 | 0.12 | 189.7 | 9.14 | 98.33 | 10.12 | 0.182 | 0.06 | 21,3 | 9,8 | 45.3 | 23.9 |
| N | 16.96 | 0.10 | 17.31 | 1.22 | **189.5** | 11.9 | 10 | 0.15 | **238.3** | 11.95 | 119.33 | 11.68 | **0.31** | 0.17 | 87,5 | 82,7 | 141 | 127 |
| H | 17.06 | 0.05 | 15.64 | 0.19 | 173.3 | 1.70 | 10.4 | 0.08 | 211.7 | 16.65 | 105.67 | 7.77 | 0.158 | 0.04 | 35,7 | 13,3 | 59.3 | 23.0 |
| I | 17.01 | 0.08 | 16.24 | 0.67 | 176.7 | 10 | 10.3 | 0.33 | 232.3 | 30.24 | 116 | 15.39 | 0.283 | 0.15 | 83,8 | 49,9 | 140 | 87.2 |
| H-N | 16.99 | 0.16 | 17.6 | 0.96 | 192.8 | 6.90 | 10.3 | 0.03 | 243.3 | 13.65 | 243.33 | 6.00 | 0.272 | 0.06 | 65,0 | 19,6 | 129 | 32.6 |
| I-N | 17.02 | 0.06 | 18.65 | 0.80 | 203.2 | 5.70 | 10.6 | 0.06 | 249.7 | 23.12 | 125 | 11.27 | 0.248 | 0.08 | 92,4 | 44,2 | 150 | 70.6 |
| I-H | 17.02 | 0.08 | 14.36 | 1.12 | 159.8 | 14.4 | 9.99 | 0.24 | 206 | 24.27 | 102.67 | 12.42 | 0.23 | 0.04 | 24,1 | 11,4 | 44.2 | 27.5 |
| I-H-N | 17.04 | 0.23 | 18.64 | 0.94 | 204.7 | 8.40 | 9.19 | 1.01 | 227.7 | 28.36 | 114 | 13.89 | 0.281 | 0.1 | 65,3 | 34,8 | 123 | 66.9 |
|  |  |  |  |  |  |  |  |  |  |  |  |  |  |  |  |  |  |  |
| D7 | | | | | | | | | | | | | | | | | | |
|  | Temp. (°C) | | DO (mg/L) | | DO (% sat.) | | pH (-) | | EC (µs/cm) | | TDS (ppm) | | TIN (mg/L) | | P-PO4 (µg/L) | | TP (µg/L) | |
|  | mean | s.d. | mean | s.d. | mean | s.d. | mean | s.d. | mean | s.d. | mean | s.d. | mean | s.d. | mean | s.d. | mean | s.d. |
| C | 21.95 | 0.09 | 12.74 | 0.31 | 152.13 | 2.58 | 9.83 | 0.17 | 202.7 | 16.01 | 101 | 8.00 | 0.201 | 0.02 | 16,1 | 5,9 | 46.2 | 3.65 |
| N | 21.77 | 0.27 | 14.96 | 0.93 | **182.3** | 10.51 | **10.1** | 0.10 | **261.7** | 10.51 | 164 | 55.97 | **1.16** | 0.17 | **206,2** | 66,0 | **268** | 116 |
| H | 22.21 | 0.42 | 7.12 | 0.28 | **88.77** | 4.12 | **9.02** | 0.04 | 193.7 | 72.6 | 113.67 | 7.77 | 0.194 | 0.01 | 50,5 | 29,7 | 50.2 | 27.9 |
| I | 21.87 | 0.11 | 11.94 | 0.80 | 146.67 | 11.03 | 10.1 | 0.29 | 242 | 38.51 | 154.67 | 74.57 | 0.3 | 0.08 | 51,8 | 24,1 | 88.8 | 41.0 |
| H-N | 21.65 | 0.36 | 9.19 | 0.51 | 112.23 | 6.26 | 9.26 | 0.09 | 254.3 | 11.85 | 254.33 | 0.58 | 1.26 | 0.18 | 280,9 | 99,1 | 388 | 166 |
| I-N | 21.74 | 0.12 | 15.81 | 0.90 | 190.27 | 9.65 | 10.6 | 0.03 | 266.3 | 23.86 | 128.33 | 20.31 | 0.965 | 0.11 | 206,1 | 59,3 | 278 | 92.4 |
| I-H | 22.00 | 0.12 | 7.45 | 0.76 | 90.97 | 10.26 | **8.64** | 0.37 | 229.3 | 16.17 | 114.67 | 8.08 | 0.221 | 0.01 | 17,1 | 15,7 | 37.3 | 17.6 |
| I-H-N | 21.91 | 0.06 | 9.28 | 0.56 | 113.1 | 6.60 | 9.17 | 0.07 | 243 | 23.26 | 121.67 | 11.68 | 1.1 | 0.05 | 154,8 | 54,1 | 238 | 82.4 |
|  |  |  |  |  |  |  |  |  |  |  |  |  |  |  |  |  |  |  |
| D14 | | | | | | | | | | | | | | | | | | |
|  | Temp. (°C) | | DO (mg/L) | | DO (% sat.) | | pH (-) | | EC (µs/cm) | | TDS (ppm) | | TIN (mg/L) | | P-PO4 (µg/L) | | TP (µg/L) | |
|  | mean | s.d. | mean | s.d. | mean | s.d. | mean | s.d. | mean | s.d. | mean | s.d. | mean | s.d. | mean | s.d. | mean | s.d. |
| C | 18.65 | 0.19 | 12.39 | 0.8 | 142.77 | 9.03 | 10.1 | 0.08 | 210 | 13.23 | 105 | 6.24 | 0.154 | 0.03 | 22,6 | 15,3 | 51.2 | 24 |
| N | 18.56 | 0.14 | 13.06 | 0.78 | 150.23 | 7.05 | 10.1 | 0.02 | **256** | 7.05 | 128 | 7.55 | **1.21** | 0.05 | **259,0** | 94,4 | **312** | 125 |
| H | 18.5 | 0.15 | 9.27 | 0.76 | **106.53** | 8.2 | **8.8** | 0.16 | **260.3** | 14.36 | 128.33 | 9.87 | 0.203 | 0.06 | 99,3 | 50,4 | 126 | 66.5 |
| I | 18.41 | 0.26 | 12.22 | 0.42 | 139.27 | 6.14 | **10.1** | 0.31 | 245.7 | 30.14 | 122.67 | 15.63 | 0.166 | 0.05 | 38,0 | 14,9 | 69.4 | 19.5 |
| H-N | 18.54 | 0.27 | 8.11 | 1.5 | 93.17 | 16.87 | 8.53 | 0.58 | 298.3 | 25.48 | 298.33 | 12.5 | 1.55 | 0.23 | 348,5 | 82,6 | 409 | 98 |
| I-N | 18.53 | 0.13 | 12.85 | 0.71 | **147.13** | 8.45 | 10.3 | 0.26 | 261.3 | 17.67 | 130.67 | 9.29 | 1.17 | 0.16 | 257,3 | 125,7 | 315 | 163 |
| I-H | 18.58 | 0.14 | 8.82 | 0.89 | 100.83 | 11.81 | 8.87 | 0.14 | **253.7** | 19.22 | 126.67 | 9.07 | 0.178 | 0.01 | 17,6 | 7,2 | 46.4 | 13.6 |
| I-H-N | 18.56 | 0.08 | 10.78 | 0.91 | **124.63** | 10.11 | 9.39 | 0.25 | 258 | 28.62 | 196 | 46.68 | 1.35 | 0.04 | 190,6 | 104,2 | 273 | 86.2 |

**Table S4**. Results of generalized linear mixed models for the *D.pulex* **juveniles**. I: insecticide =chlorpyrifos, H: herbicide=diuron, P: predation, $\hat{\boldsymbol{\beta}}$ = Estimated effect, S.E. = standard error, “x” indicates interactions. Bold values indicate significant effects of the individual stressor or the stressor interactions (P-value < 0.05).

|  |  | **Mesotrophic** | | |  | **Eutrophic** | | |
| --- | --- | --- | --- | --- | --- | --- | --- | --- |
| **Days** |  | **Fixed Effects** | $\hat{\boldsymbol{\beta}}$ **(± S.E.)** | **P-value** |  | **Fixed Effects** | $\hat{\boldsymbol{\beta}}$ **(± S.E.)** | **P-value** |
| **D 2** |  | **Constant**  **I** | **4.532 ± 0.135**  **-3.472 ± 0.374** | **< 0.001**  **< 0.001** |  | **Constant**  **I** | **3.595 ± 0.434**  **-3.576 ± 0.772** | **< 0.001**  **< 0.001** |
|  |  | H | -0.373 ± 0.194 | 0.054 |  | H | 0.706 ± 0.601 | 0.240 |
|  |  | **P** | **-1.373 ± 0.131** | **< 0.001** |  | **P** | **-2.403 ± 0.290** | **< 0.001** |
|  |  | IxH | -0.902 ± 0.728 | 0.215 |  | IxH | -0.441 ± 1.043 | 0.673 |
|  |  | IxP | -0.454 ± 0.846 | 0.592 |  | IxP | 1.707 ± 0.883 | 0.053 |
|  |  | **HxP** | **-1.110 ± 0.286** | **< 0.001** |  | **HxP** | **0.825 ± 0.315** | **0.009** |
|  |  | **IxHxP** | **3.761 ± 1.105** | **< 0.001** |  | IxHxP | -0.616 ± 1.098 | 0.575 |
|  |  |  |  |  |  |  |  |  |
| **D 7** |  | **Constant**  **I** | **3.867 ± 0.505**  **-2.772 ± 0.762** | **< 0.001**  **< 0.001** |  | **Constant**  **I** | **3.305 ± 0.459**  **-2.974 ± 0.734** | **< 0.001**  **< 0.001** |
|  |  | **H** | **-1.466 ± 0.716** | **0.041** |  | H | 0.716 ± 0.635 | 0.259 |
|  |  | **P** | **-0.509 ± 0.119** | **< 0.001** |  | **P** | **-1.712 ± 0.247** | **< 0.001** |
|  |  | IxH | 1.042 ± 1.071 | 0.331 |  | IxH | 0.265 ± 0.989 | 0.789 |
|  |  | IxP | 0.441 ± 0.481 | 0.359 |  | **IxP** | **3.119 ± 0.476** | **< 0.001** |
|  |  | **HxP** | **-2.506 ± 0.477** | **< 0.001** |  | **HxP** | **0.815 ± 0.281** | **0.004** |
|  |  | **IxHxP** | **4.380 ± 0.772** | **< 0.001** |  | **IxHxP** | **-3.130 ± 0.670** | **< 0.001** |
|  |  |  |  |  |  |  |  |  |
| **D14** |  | **Constant**  I | **3.006 ± 0.498**  1.150 ± 0.689 | **< 0.001**  0.095 |  | **Constant**  **I** | **3.933 ± 0.197**  **-1.786 ± 0.327** | **< 0.001**  **< 0.001** |
|  |  | H | -0.907 ± 0.709 | 0.201 |  | **H** | **-0.750 ± 0.289** | **0.010** |
|  |  | **P** | **0.528 ± 0.153** | **< 0.001** |  | **P** | **-0.692 ± 0.134** | **< 0.001** |
|  |  | IxH | -0.202 ± 0.979 | 0.837 |  | **IxH** | **2.912 ± 0.432** | **< 0.001** |
|  |  | IxP | 0.139 ± 0.171 | 0.415 |  | **IxP** | **2.017 ± 0.253** | **< 0.001** |
|  |  | HxP | -0.284 ± 0.289 | 0.327 |  | **HxP** | **0.721 ± 0.207** | **< 0.001** |
|  |  | **IxHxP** | **-1.116 ± 0.339** | **< 0.001** |  | **IxHxP** | **-0.927 ± 0.307** | **0.002** |
|  |  |  |  |  |  |  |  |  |
| **D21** |  | **Constant**  I | **2.712 ± 0.401**  -0.183 ± 0.561 | **< 0.001**  0.744 |  | **Constant**  I | **4.399 ± 0.326**  -0.062 ± 0.458 | **< 0.001**  0.892 |
|  |  | H | -1.160 ± 0.598 | 0.052 |  | H | -0.609 ± 0.461 | 0.187 |
|  |  | **P** | **0.416 ± 0.179** | **0.020** |  | **P** | **-1.313 ± 0.133** | **< 0.001** |
|  |  | IxH | 0.507 ± 0.824 | 0.539 |  | IxH | 0.069 ± 0.647 | 0.916 |
|  |  | **IxP** | **1.185 ± 0.227** | **< 0.001** |  | **IxP** | **1.676 ± 0.154** | **< 0.001** |
|  |  | **HxP** | **-1.858 ± 0.543** | **< 0.001** |  | **HxP** | **1.468 ± 0.177** | **< 0.001** |
|  |  | IxHxP | 0.578 ± 0.622 | 0.352 |  | **IxHxP** | **-0.538 ± 0.210** | **0.011** |
|  |  |  |  |  |  |  |  |  |

**Table S5**. Results of generalized linear mixed models for the *D. pulex* **adults**. I: insecticide =chlorpyrifos, H: herbicide=diuron, P: predation, $\hat{\boldsymbol{\beta}}$ = Estimated effect, S.E. = standard error. “x” indicates interactions. Bold values indicate significant effects of the individual stressor or the stressor interactions (P-value < 0.05).

|  |  | **Mesotrophic** | | |  | **Eutrophic** | | |
| --- | --- | --- | --- | --- | --- | --- | --- | --- |
| **Days** |  | **Fixed Effects** | $\hat{\boldsymbol{\beta}}$ **(± S.E.)** | **P-value** |  | **Fixed Effects** | $\hat{\boldsymbol{\beta}}$ **(± S.E.)** | **P-value** |
| D 2 |  | **Constant**  **I** | **3.464 ± 0.372**  **-4.477 ± 0.957** | **< 0.001**  **< 0.001** |  | **Constant**  **I** | **2.744 ± 0.242**  **-4.978 ± 1.431** | **< 0.001**  **< 0.001** |
|  |  | H | -0.114 ± 0.519 | 0.827 |  | **H** | **0.762 ± 0.322** | **0.018** |
|  |  | **P** | **-4.382 ± 0.779** | **< 0.001** |  | **P** | **-4.347 ± 1.092** | **< 0.001** |
|  |  | IxH | -1.834 ± 2.004 | 0.360 |  | IxH | -1.592 ± 2.163 | 0.462 |
|  |  | IxP | -0.160 ± 2.940 | 0.957 |  | IxP | -0.159 ± 2.962 | 0.957 |
|  |  | HxP | 0.365 ± 1.020 | 0.720 |  | HxP | 2.140 ± 1.124 | 0.057 |
|  |  | IxHxP | -0.027 ± 3.122 | 0.993 |  | IxHxP | -0.122 ± 3.009 | 0.968 |
|  |  |  |  |  |  |  |  |  |
| D 7 |  | **Constant**  **I** | **1.495 ± 0.415**  **-3.241 ± 1.187** | **< 0.001**  **0.006** |  | **Constant**  **I** | **1.458 ± 0.378**  **-1.402 ± 0.654** | **< 0.001**  **0.032** |
|  |  | H | -1.029 ± 0.682 | 0.131 |  | H | 0.644 ± 0.474 | 0.175 |
|  |  | **P** | **-3.251 ± 1.132** | **0.004** |  | **P** | **-3.307 ± 1.147** | **0.004** |
|  |  | IxH | -1.007 ± 2.343 | 0.667 |  | IxH | -0.529 ± 0.891 | 0.553 |
|  |  | IxP | 3.349 ± 1.725 | 0.052 |  | **IxP** | **3.006 ± 1.315** | **0.022** |
|  |  | HxP | -1.044 ± 2.307 | 0.651 |  | HxP | -2.175 ± 2.033 | 0.285 |
|  |  | IxHxP | -0.236 ± 2.910 | 0.935 |  | IxHxP | -1.102 ± 2.451 | 0.653 |
|  |  |  |  |  |  |  |  |  |
| D14 |  | **Constant**  **I** | **1.122 ± 0.354**  **1.359 ± 0.419** | **0.002**  **0.001** |  | **Constant**  I | **2.285 ± 0.436**  -0.773 ± 0.632 | **< 0.001**  0.221 |
|  |  | **H** | **1.062 ± 0.428** | **0.013** |  | H | 0.896 ± 0.597 | 0.133 |
|  |  | **P** | **-1.286 ± 0.634** | **0.043** |  | P | 0.125 ± 0.231 | 0.590 |
|  |  | **IxH** | **-1.217 ± 0.539** | **0.024** |  | IxH | -0.473 ± 0.866 | 0.585 |
|  |  | **IxP** | **1.398 ± 0.666** | **0.036** |  | **IxP** | **0.863 ± 0.352** | **0.014** |
|  |  | HxP | 0.974 ± 0.686 | 0.156 |  | HxP | 0.070 ± 0.262 | 0.788 |
|  |  | **IxHxP** | **-2.881 ± 0.839** | **< 0.001** |  | IxHxP | -0.744 ± 0.461 | 0.106 |
|  |  |  |  |  |  |  |  |  |
| D21 |  | Constant  **I** | **0.423 ± 0.442**  **1.264 ± 0.500** | **0.339**  **0.011** |  | **Constant**  **I** | **2.894 ± 0.308**  **0.994 ± 0.421** | **< 0.001**  **0.018** |
|  |  | H | -2.420 ± 1.258 | 0.054 |  | H | 0.041 ± 0.432 | 0.925 |
|  |  | P | -0.418 ± 0.689 | 0.544 |  | **P** | **-0.682 ± 0.220** | **0.002** |
|  |  | IxH | 2.509 ± 1.284 | 0.051 |  | IxH | -0.131 ± 0.592 | 0.825 |
|  |  | IxP | 0.573 ± 0.755 | 0.448 |  | IxP | 0.479 ± 0.246 | 0.052 |
|  |  | HxP | -1.137 ± 1.927 | 0.555 |  | HxP | 0.309 ± 0.298 | 0.299 |
|  |  | IxHxP | -0.277 ± 1.949 | 0.887 |  | IxHxP | -0.112 ± 0.337 | 0.738 |

**Table S6**. *D. pulex* adults and juveniles abundance in the different treatments under (A) mesotrophic and (B) eutrophic conditions. C: control, I: insecticide, H: herbicide, P: predation. D0-D21 refer to the different sampling days.

| **A. Mesotrophic** | C | | I | | H | | I-H | |
| --- | --- | --- | --- | --- | --- | --- | --- | --- |
|  | Adults | Juveniles | Adults | Juveniles | Adults | Juveniles | Adults | Juveniles |
| D0 | 50.0 | 50.0 | 50.0 | 50.0 | 50.0 | 50.0 | 50.0 | 50.0 |
| D2 | 39.3 | 95.7 | 0.3 | 3.0 | 36.3 | 66.0 | 0.0 | 0.7 |
| D7 | 5.3 | 63.0 | 0.0 | 3.0 | 2.0 | 32.0 | 0.0 | 2.0 |
| D14 | 3.3 | 22.3 | 12.3 | 84.3 | 9.3 | 9.3 | 10.7 | 39.7 |
| D21 | 1.7 | 17.0 | 5.3 | 20.0 | 0.0 | 6.0 | 6.0 | 7.0 |
|  | P | | I-P | | H-P | | I-H-P | |
|  | Adults | Juveniles | Adults | Juveniles | Adults | Juveniles | Adults | Juveniles |
| D0 | 50.0 | 50.0 | 50.0 | 50.0 | 50.0 | 50.0 | 50.0 | 50.0 |
| D2 | 0.3 | 24.3 | 0.0 | 0.3 | 0.7 | 5.3 | 0.0 | 2.0 |
| D7 | 0.0 | 38.0 | 0.3 | 2.7 | 0.0 | 1.3 | 0.0 | 13.0 |
| D14 | 0.7 | 37.7 | 14.0 | 164.3 | 7.0 | 12.0 | 1.7 | 19.0 |
| D21 | 1.0 | 25.7 | 6.3 | 99.3 | 0.0 | 1.3 | 1.7 | 9.7 |
|  |  |  |  |  |  |  |  |  |
| **B. Eutrophic** | C | | I | | H | | I-H | |
|  | Adults | Juveniles | Adults | Juveniles | Adults | Juveniles | Adults | Juveniles |
| D0 | 50.0 | 50.0 | 50.0 | 50.0 | 50.0 | 50.0 | 50.0 | 50.0 |
| D2 | 17.0 | 46.7 | 0.0 | 1.0 | 35.7 | 127.3 | 0.0 | 1.7 |
| D7 | 5.0 | 35.3 | 1.0 | 2.0 | 8.7 | 61.7 | 1.3 | 5.3 |
| D14 | 11.3 | 55.3 | 6.0 | 8.7 | 38.7 | 25.7 | 7.3 | 76.3 |
| D21 | 20.0 | 87.7 | 58.0 | 96.3 | 19.3 | 44.7 | 52.0 | 60.7 |
|  | P | | I-P | | H-P | | I-H-P | |
|  | Adults | Juveniles | Adults | Juveniles | Adults | Juveniles | Adults | Juveniles |
| D0 | 50.0 | 50.0 | 50.0 | 50.0 | 50.0 | 50.0 | 50.0 | 50.0 |
| D2 | 0.0 | 4.0 | 0.0 | 0.7 | 4.0 | 26.3 | 0.0 | 1.0 |
| D7 | 0.0 | 6.0 | 1.0 | 9.7 | 0.0 | 25.3 | 0.0 | 2.0 |
| D14 | 12.7 | 27.3 | 16.3 | 33.7 | 47.0 | 26.7 | 10.0 | 233.3 |
| D21 | 10.0 | 23.3 | 47.3 | 138.7 | 13.3 | 52.3 | 51.7 | 221.0 |
